# Respiratory function in adult patients with spinal muscular atrophy treated with nusinersen – a monocenter observational study

**DOI:** 10.3389/fneur.2024.1372674

**Published:** 2024-04-03

**Authors:** Claudia Diana Wurster, Zeljko Uzelac, Jens Dreyhaupt, Joachim Schuster, Johannes Dorst, Albert Christian Ludolph, Kurt Wollinsky

**Affiliations:** ^1^Department of Neurology, Ulm University, Ulm, Germany; ^2^Institute of Human Genetics, Ulm University Medical Center, Ulm, Germany; ^3^Department of Epidemiology and Medical Biometry, Ulm University, Ulm, Germany; ^4^German Center for Neurodegenerative Diseases (DZNE), Ulm, Germany; ^5^Department of Anesthesiology, RKU, Ulm University, Ulm, Germany

**Keywords:** spinal muscular atrophy (SMA), nusinersen, respiratory function, forced vital capacity, adult patients

## Abstract

**Background/objective:**

Insufficiency of respiratory muscles is the most important reason for mortality in the natural history of SMA. Thus, improvement or stabilization of respiratory function by disease-modifying therapies (DMT) is a very important issue.

**Methods:**

We examined respiratory function using forced vital capacity (FVC) in 42 adult SMA patients (2 SMA type 1, 15 SMA type 2, 24 SMA type 3, 1 SMA type 4, median age 37 years, range 17–61 years) treated with nusinersen for a median of 22.1 months (range 2.1 to 46.7 months). Change in FVC was assessed using mixed effects linear regression models.

**Results:**

Baseline FVC differed significantly between SMA type 1 (4.0, 8.0%), 2 (median 22.0%, IQR 18.0–44.0), 3 (median 81.0%, IQR 67.0–90.8) and, respectively, type 4 (84.0%) patients reflecting the heterogeneity of respiratory impairment based on the SMA type in adulthood (*p* < 0.0001). FVC remained stable during follow-up (mean −0.047, 95% CI −0.115 to 0.020, *p* = 0.17); however, subgroup analysis showed an increase in FVC of type 2 patients (mean 0.144, 95% CI 0.086 to 0.202, *p* < 0.0001) and a decrease in FVC of type 3/4 patients (−0.142, 95% CI −0.239 to −0.044, *p* = 0.005).

**Conclusion:**

The observed improvement in FVC in patients with SMA type 2 can be seen as a therapeutic response differing from the progressive decline typically seen in the spontaneous course. For SMA type 3/4 patients approaching normal spirometry at baseline, FVC may only be of limited use as an outcome parameter due to ceiling effects.

## 1 Introduction

Spinal muscular atrophy (SMA) is a neuromuscular disease caused by a homozygous deletion or mutation in the survival of motoneuron- (*SMN1*) gene (1, 2), leading to degeneration of alpha-motoneurons and thus to progressive muscular weakness.

The clinical phenotype of patients comprises a wide spectrum from relatively mild to extremely severe forms. Until now, SMA patients are classified as different SMA types (0–4) depending on age at disease onset, the ability to achieve motor milestones and survival (3). A new classification based on the patients’ current function is becoming more frequently used (non-sitter, sitter, walker) (4, 5).

Insufficiency of respiratory muscles is the most important reason for mortality during the spontaneous course of disease in SMA type 1 and 2, rarely also type 3 (6, 7). Intercostal muscles are more affected than the diaphragm muscle, clinically demonstrated by the paradoxical breathing pattern (“belly-breathing”) and a bell-shaped chest (8, 9). However, the restrictive ventilatory disorder in SMA is not only caused by weakness of the respiratory muscles, but often further complicated by severe scoliosis and contractures leading to deformation and constriction of thorax and lungs. Growth and development of these structures are limited in severely affected SMA patients. Secretion retention and aspiration cause acute and chronic respiratory infections, often leading to dys-and atelectasis and further exacerbation of restriction (10).

Thus, improvement or stabilization of respiratory function in SMA is a very important issue. The antisense-oligonucleotide nusinersen became the first disease-modifying drug to be approved by the FDA in 2016 and the EMA in 2017. The ENDEAR trial showed that the probability of survival and/or not needing permanent ventilation in SMA type 1 was significantly higher in the nusinersen group than in the placebo group (11), yet unmet respiratory needs for these patients remain as real-life data show (12, 13). No respiratory data were collected for later-onset SMA (type 2 and 3) in the placebo-controlled pivotal trial (CHERISH) (14), and no adults were enrolled in these studies. Only few post-approval studies have examined respiratory changes during nusinersen treatment in adult SMA patients so far (15–18). Here, we focus on changes in respiratory function measured by forced vital capacity (FVC) in adult SMA patients treated with nusinersen.

## 2 Materials and methods

### 2.1 Study design and participants

SMA patients were enrolled at the Department of Neurology, Ulm University Hospital between June 2017 and October 2021. The prospective, observational study was approved by the local ethics committee in Ulm (approval number University of Ulm 19/12; 2012; MND registry) and all patients gave informed written consent to participate in the study. All patients had genetically confirmed 5q-SMA (deletion (or mutation) in exon 7 and/or 8 of the *SMN1* gene). Patients’ history and clinical data were collected before therapy started (Table 1). SMA type classification was based on the onset of disease and motor skills achieved. Patients received intrathecal administrations of 12 mg nusinersen following the standard of care dosing schedule (treatment day 0, 14, 28 and 63 and every 4 months). Intrathecal injection of nusinersen was performed by standard access; for patients with severe scoliosis a CT-guided lumbar puncture was carried out (19).

**Table 1:** Demographics and baseline functional assessment.

	Total	SMA type 1	SMA type 2	SMA type 3	SMA type 4
n	42 (100.0%)	2 (4.8%)	15 (35.7%)	24 (57.1%)	1 (2.4%)
Female	19	1	9	9	0
Male	23	1	6	15	1
Age (years)	37.0, 24.8–49.3 (17.0–61.0)	18.0, 23.0	29.0, 21.0–45.0 (18.0–51.0)	42.5, 34.3–51.8 (17.0–61.0)	51.0
Disease onset (years)	1.0, 0.0–7.8 (0.0–36.0)	0.0, 0.0	0.0, 0.0–1.0 (0.0–1.0)	4.5, 1.3–14.5 (0.0–30.0)	36.0
Ambulatory	10	0	0	9	1
BMI	23.2, 19.1–27.6 (8.1–42.3)	19.4, 18.6	19.2, 16.8–23.3 (8.1–27.5)	24.8, 22.6–30.2 (15.8–42.3)	26.6
PEG/NG tube	4	1	3	0	0
Scoliosis	25	2	15	8	0
Spinal surgery	11	0	7	4	0
NIV	18	2	13	3	0
Smoking	9	0	0	8	1
HFMSE (T1)	7.5, 0.0–37.8 (0.0–66.0)		0.0, 0.0–2.0 (0.0–8.0)	25.0, 8.5–55.5 (4.0–66.0)	61.0
*SMN2* copies	3 *SMN2*: 22 4 *SMN2*: 16 (*missing: 4*)	3 *SMN2*: 1 4 *SMN2*: 0 (*missing: 1*)	3 *SMN2*: 10 4 *SMN2*: 4 (*missing: 1*)	3 *SMN2*: 11 4 *SMN2*: 11 (*missing: 2*)	3 *SMN2*: 0 4 *SMN2*: 1 (*missing: 0*)
FVC baseline (T1)	65.5, 23.5–84.8 (4.0–118.0)	4.0, 8.0	22.0, 18.0–44.0 (11.0–66.0)	81.0, 67.0–90.8 (28.0–118.0)	84.0

Categorical data are described as frequencies (percentage (%)); continuous data by median, quartiles (IQR) and range (in brackets).

SMA, spinal muscular atrophy; BMI, Body Mass Index; PEG, percutaneous endoscopic gastrostomy; NG tube, nasogastric tube; NIV, Non-invasive ventilation; h, hours; HFMSE, Hammersmith Functional Motor Score Expanded; T1, baseline (treatment day 0); SMN2, survival of motoneuron 2 (gene copies); FVC, forced vital capacity.

Over time, the number of measurements decreased due to the following circumstances: first, start of treatment varied among patients. Second, there were few drop-outs: two patients continued therapy at a center close to home and two patients opted to switch therapy. In addition, spirometry was briefly paused for all patients to avoid aerosol exposure at the beginning of the corona pandemic (SARS-CoV-2, COVID-19).

### 2.2 Spirometry

Spirometry was performed along with the intrathecal administration of nusinersen on treatment days 0 (baseline, T1), 63 (T4) and every 4 months (T5-T15). FVC was measured either in sitting or in lying position. Patients wore a nose clip while performing the breathing maneuvers; the hand manometer (ProSpiro mobile edition, MESA Medizintechnik GmbH, Benediktbeuren, Germany) was either held by the patients themselves, or it was held for them if they were unable due to their muscular weakness. Patients performed at least three breathing maneuvers to determine FVC, with the best attempt scored. Usually, the same experienced anesthesiologist performed the examination.

### 2.3 Statistics

Linear mixed models were used to explore the change of FVC across time with random intercepts. Results were reported as mean difference and 95% confidence intervals (CI). Positive values indicate increase compared to baseline and negative values indicate decrease from baseline. Continuous data were described by median (quartiles, IQR) and range, categorical data as frequencies (percentage (%)).

The associations between FVC and motor function measured by the Hammersmith Functional Motor Scale Expanded (HFMSE) (20) were analyzed by scatter plot and Spearman’s rank correlation coefficient.

Subgroup analysis included two groups: SMA type 2 patients and SMA type 3 patients, with the only type 4 patient assigned to the SMA type 3 group. The SMA type 3 subgroup was divided into a smoker versus non-smoker subgroup for a further analysis.

Because of the explorative character of this study, the results of the statistical tests were not interpreted as confirmatory but as hypothesis generating only. Accordingly, no adjustment for multiple testing was done. A two-sided *p*-value <0.05 was interpreted as statistically significant. Statistical analysis was performed with the software GraphPad Prism 9 and SAS 9.4 under Windows.

## 3 Results

Forty-two patients (45% female, 55% male), median age 37 years, range between 17 and 61 years, were enrolled. Two patients were classified as SMA type 1, 15 as SMA type 2, 24 as SMA type 3 and one as SMA type 4.

Both SMA type 1 patients required non-invasive ventilation (NIV) (8 h/24 h and 18 h/24 h, respectively); 13 of 15 SMA type 2 patients used NIV overnight (approx. 8 h/24 h); 3 of 24 SMA type 3 patients had a ventilator (CPAP, respectively, NIV), with two of these patients having an additional diagnosis of OSAS, and the third patient using the ventilator only when needed (e.g., during an infection). Eight of the SMA type 3 patients and the SMA type 4 patient reported daily smoking, whereas none of the SMA type 1 or 2 patients did so.

Detailed baseline characteristics are shown in Table 1.

FVC differed significantly between the SMA types at baseline (*p* < 0.0001). While the two SMA type 1 patients showed FVC values of 4% and 8%, type 2 patients had median values of 22% (IQR 18–44%), and type 3/4 patients’ median values of 81% (IQR 67–91%) and 84%, respectively (Table 1).

Patients were treated with nusinersen for a median time of 22.1 months (range 2.1 to 46.7 months). Mixed regression analysis showed that the FVC of the entire study population did not change significantly during treatment (*p* = 0.17). The rate of change in FVC for all SMA patients over time was −0.047 (95% CI −0.115 to 0.020) per month.

**Figure 1:**
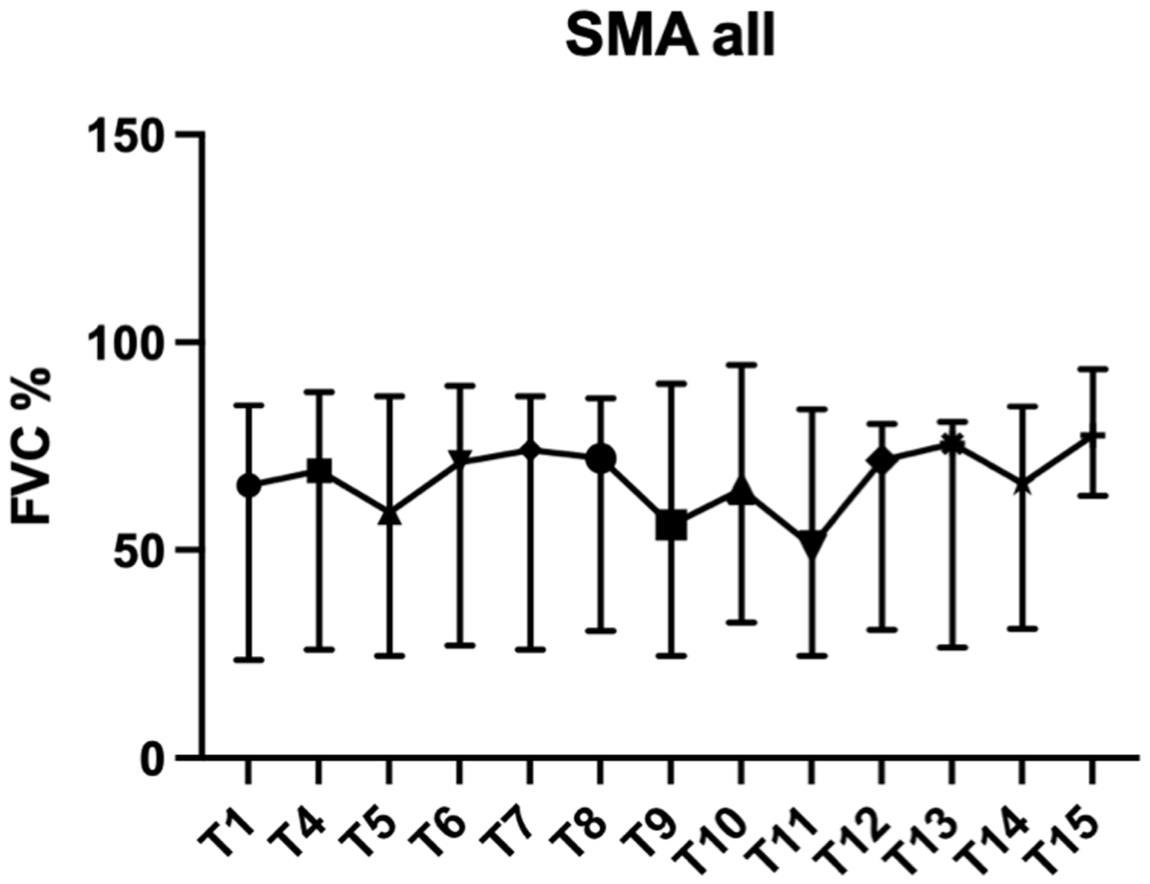
FVC% SMA all: values are in median and IQR.

**Figure 2:**
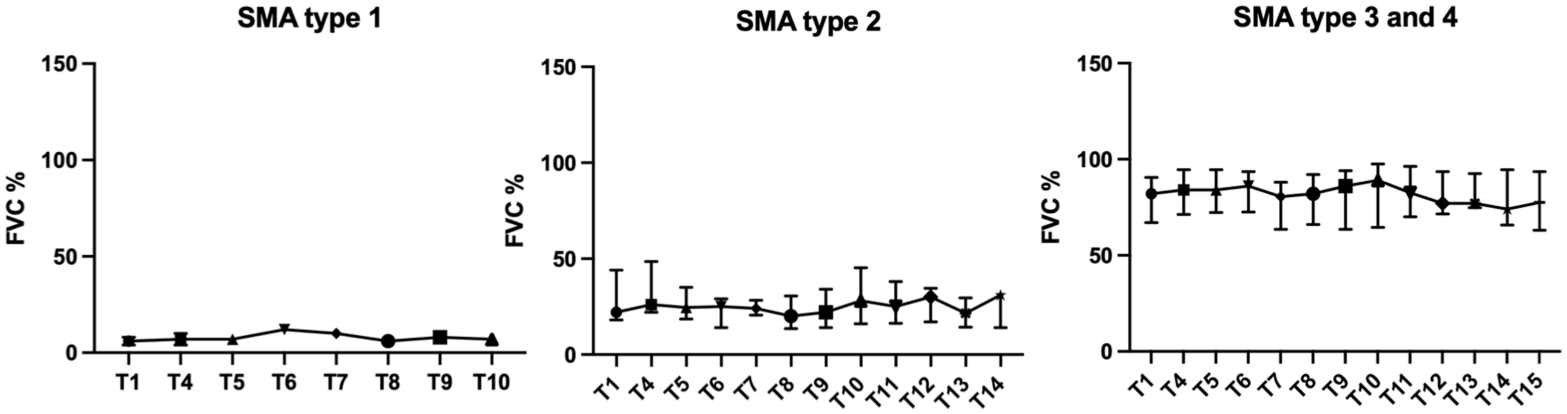
FVC% SMA subtypes: values are in median and IQR.

Subgroup analysis by SMA type (type 2 and type 3/4), showed significant, but opposite changes in FVC. In SMA type 2 patients, FVC increased over time (0.144 per month, 95% CI 0.086 to 0.202, *p* < 0.0001), whereas FVC of SMA type 3/4 patients decreased over time (−0.142 per month, 95% CI 0.239 to −0.044, *p* = 0.005) (Figures 1, 2). Further analysis for the SMA type 3 smokers showed the strongest decrease (−0.256 per month, 95% CI −0.388 to −0.124, *p* = 0.0003), the SMA type 3 non-smokers had a clearly less strong change (−0.066 per month, 95% CI −0.204 to 0.072, *p* = 0.34).

The scatter plot showed no recognizable association between the individual slopes of FVC and HFMSE (not shown). The correlation coefficient according to Spearman was −0.14 (*p* = 0.51).

## 4 Discussion

We found that FVC of adult SMA patients did not change significantly under nusinersen therapy. However, subgroup analysis showed an improvement of FVC in SMA type 2 and a worsening in SMA type 3/4 patients.

Natural history of respiratory function in SMA shows that deterioration of pulmonary function is similar to decline of motor function. Since respiratory function in the acute, infantile form (SMA type 1) already deteriorates massively at a very early stage in life, surveys of FVC are usually not available for this patient group and are limited to the chronic forms of progression (SMA types 1c to 4). In general, decrease of FVC in SMA type 1c to 3a is greatest in childhood, with a slower decline or stabilization during early adulthood. In SMA type 3b and 4, FVC seems to remain relatively stable during the course of disease (21). Annual rates of FVC decline in untreated SMA type 2 and 3 patients were reported by-1.13% *per annum* (22) respectively between −0.2% and −1.4% (21). Children and young adults with SMA type 2 have a greater drop in FVC than SMA type 3 patients (23, 24). Accordingly, FVC values are expected to diverge significantly between untreated SMA phenotypes. Wijngaarde et al. showed differences in FVC varying from 50% in SMA type 1c, 64% in SMA type 2a, 85% in SMA type 2b, 97% in SMA type 3a, and 109% in SMA type 3b (21). Chabanon et al. demonstrated that FVC significantly separated four functional groups with the lowest values in SMA type 2 non-sitter (44%), followed by SMA type 2 sitter (62%), SMA type 3 non-walkers (90%) and SMA type 3 walkers (96%), which in general showed a normal respiratory function (*n* = 43) (25). Both children and adult SMA patients were included in these analyses (aged 4 to 74 years, Wijngaarde et al.; aged 2 to 30 years, Chabanon et al.). Our data show that in adulthood, FVC is worse than previous data had suggested and values between SMA phenotypes vary even more.

Real-world data examining the efficacy of nusinersen on respiratory function in children with SMA types 2 and 3 seemed to show contradictory results. A significant improvement of FVC in children with later-onset SMA compared to historical controls was demonstrated by a French study (26). In contrast, a German study concluded that FVC remains stable and does not improve during nusinersen therapy in children (27). Although both studies are comparable regarding patient numbers (*n* = 12), age and observation period (14 and 10 months after treatment initiation, respectively), it should be noted that the latter study included predominantly ambulatory SMA type 3 patients, who are expected to have an unaffected FVC. The French study instead focused on children with SMA type 2, which could explain the differences. Another study did not show any improvement, but at least a slower decline in FVC during the first year of nusinersen treatment, particularly in SMA type 2 children (28).

Similar results have been published for cohorts of adult SMA patients treated with nusinersen. Most studies show unchanged FVC in adult SMA patients treated with nusinersen (15, 16, 18, 29, 30). Also here, a study detected an increase in FVC in ambulatory SMA patients with normal FVC at the start of therapy, so the clinical impact remains unclear (31). In accordance to our results, an American study demonstrated a relatively stable (annual) FVC rate of 0.75 in adult SMA patients treated with nusinersen, whereas subgroup analysis showed an improvement with a slope of 2.61 for SMA type 2 patients and a decrease of −0.31 for SMA type 3 patients (walker −1.97) (17).

Comparing these results to the effects of nusinersen on motor function [determined by different motor scores (e.g., HFMSE, RULM, 6MWT)] in adult SMA patients, there seems to be a discrepancy, as positive effects of therapy were reported more frequently (29, 32, 33). We observed greater improvement of motor function in treated adult SMA patients with lower severity of disease (e.g., higher proportions of patients with clinically meaningful improvements in HFMSE score in the SMA type 3 subgroup than in the SMA type 2 subgroup) in a previous study (32). However, measured effects also depend on how sensitively the respective method can depict changes. FVC, therefore, may not seem to be a valid outcome measure for adult, ambulatory SMA type 3 patients, whose respiratory function is usually normal and therefore implies a ceiling effect. Finally, it remains to be discussed why a decrease in FVC of SMA type 3 patients was found in our study. Having the clinical data of patients in mind, a high prevalence of smokers in the SMA type 3 subgroup stands out [a total of 36% of the type 3/4 patients, in comparison: 0% in type 1 and 2; German smoking prevalence: 23.8% ≥ 18 years (Federal Ministry of Health, Germany, 2021)]. To our knowledge, there is no clinical data on smokers among SMA patients; nor has a specific longitudinal analysis of FVC in adult SMA patients who smoke been carried out. Although the results must be interpreted with caution due to the small number of cases, the decrease in FVC was greatest among smokers, thus smoking could be a possible (promoting) factor.

The observed improvement in FVC in patients with SMA type 2 can be evaluated as a therapeutic response which is different from the progressive decline typically seen in the spontaneous course of disease. Follow-up studies with large patient cohorts are needed to secure the shown effects.

## Data availability statement

The raw data supporting the conclusions of this article will be made available by the authors, without undue reservation.

## Ethics statement

The studies involving humans were approved by Ethics Committee of Ulm University. The studies were conducted in accordance with the local legislation and institutional requirements. Written informed consent for participation in this study was provided by the participants’ legal guardians/next of kin.

## Author contributions

CW: Conceptualization, Data curation, Investigation, Methodology, Project administration, Writing – original draft. ZU: Conceptualization, Investigation, Writing – review & editing. JDr: Conceptualization, Data curation, Formal analysis, Software, Writing – review & editing. JS: Conceptualization, Supervision, Writing – review & editing. JDo: Conceptualization, Data curation, Supervision, Writing – review & editing. AL: Conceptualization, Methodology, Project administration, Supervision, Writing – review & editing. KW: Conceptualization, Data curation, Investigation, Methodology, Project administration, Supervision, Writing – review & editing.
